# Electroconvulsive Therapy Anesthesia Scale (ECTAS): development and preliminary expert-based evidence of content validity

**DOI:** 10.1016/j.bjane.2026.844842

**Published:** 2026-10-09

**Authors:** Matheus Veras Guterres Mendes, José Osvaldo Barbosa Neto, Gabriel Magalhães Nunes Guimarães, Bruno Bacellar Palhano, Marcelly Amanda Lucena Ericeira, Julio Cesar de Oliveira Silva, João Batista Santos Garcia

**Affiliations:** a Universidade Federal do Maranhão (UFMA), Post-Graduate Program in Adult Health, São Luís, MA, Brazil; b Universidade CEUMA, São Luís, MA, Brazil; c Universidade de Brasília, Brasília, DF, Brazil; d Hospital Universitário da Universidade Federal do Maranhão (HU-UFMA), São Luís, MA, Brazil; e Unidade de Ensino Superior Dom Bosco (UNDB), São Luís, MA, Brazil

Electroconvulsive Therapy (ECT) is an effective treatment for severe psychiatric conditions, including major depression, schizophrenia, mania, catatonia, and suicidal ideation.^1^^,^^2^ The introduction of general anesthesia has markedly improved the safety and tolerability of the procedure.^3^ Appropriate anesthetic management should provide adequate hypnosis, neuromuscular blockade, and rapid recovery.^4^

ECT is commonly performed under general anesthesia induced with an intravenous hypnotic agent followed by succinylcholine because of its rapid onset and short duration of action.^5^, ^6^, ^7^ Rocuronium followed by sugammadex represents an effective alternative; however, the additional cost associated with sugammadex may limit its routine use, particularly in resource-constrained settings.^8^ Neuromuscular monitoring can provide information regarding neuromuscular blockade,^9^ whereas assessment of the hypnotic component requires separate consideration. Consequently, bedside assessment remains part of the clinical evaluation performed before electrical stimulus delivery during ECT.

For the purposes of this study, anesthetic readiness was operationally defined as a multidomain clinical state in which bedside findings are consistent with adequate hypnotic effect and sufficient attenuation of motor response before electrical stimulation. These domains are physiologically distinct and are not intended to be interpreted as interchangeable. No standardized bedside instrument integrating these clinical observations has been established. Therefore, this study aimed to develop a structured assessment instrument and obtain preliminary expert-based evidence of content validity.

The study protocol was approved by the Research Ethics Committee of Centro Universitário do Maranhão (UNICEUMA), São Luís, Brazil (CAAE 52306021.5.0000.5084; approval n° 5.456.362; June 8, 2022). The present study reports the expert-questionnaire stage of the approved research protocol. Electronic informed consent was obtained from all participating experts before questionnaire completion. Participation was voluntary and responses were confidential. Participant names were not collected; professional e-mail addresses were collected solely to verify response uniqueness and to invite first-round respondents to participate in the second round.

Candidate items were developed from a narrative literature review (MEDLINE, SciELO, and LILACS; MeSH terms: “electroconvulsive therapy”, “anesthesia”, “anesthesiology”, “neuromuscular blockade”, and “succinylcholine”) and the authors' clinical experience. Based on the available evidence,^5^, ^6^, ^7^, ^8^, ^9^ a preliminary instrument comprising eight candidate clinical parameters was created: apparent immobility after succinylcholine-induced fasciculations (I-1); absence of eye opening to verbal commands (I-2); apnea (I-3); abolition of the corneal-palpebral reflex (I-4); reduced muscle strength (Medical Research Council grade < 2) (I-5); absent or low-intensity tonic–clonic movements (I-6); seizure duration ≥ 20 seconds (I-7); and memory of the event and/or perception of breathlessness on awakening (I-8).

Content validity was assessed using a modified Delphi process involving anesthesiologists and psychiatrists with experience in ECT. Owing to the absence of a national registry of professionals actively involved in ECT in Brazil, a non-probability convenience sampling strategy was adopted. Experts were recruited through two channels: 1) E-mail invitations sent to coordinators of four specialized ECT centers, who subsequently distributed the survey to their clinical teams; and 2) An invitation distributed via the Brazilian Society of Anesthesiology (SBA). These centers were selected because they routinely perform ECT and have established multidisciplinary teams. Because recruitment occurred indirectly through these channels, the total number of professionals who received the invitation could not be determined, precluding calculation of a response rate. Experts were eligible if they were anesthesiologists or psychiatrists with at least 2 years of self-reported clinical experience with ECT. This a priori criterion was intended to ensure sustained practical exposure to ECT while maintaining a sufficiently broad pool of eligible specialists for the preliminary Delphi process. A total of 59-first-round questionnaires were received; six incomplete questionnaires were excluded, resulting in 53 questionnaires included in the first-round analysis. No incomplete questionnaires were received in the second round. No imputation of missing data was performed.

Before definitive data collection, the questionnaire was pilot tested with a convenience sample of 10 professionals to assess its clarity and face validity. Minor wording adjustments were made, and the final version was subsequently administered electronically using Google Forms. Professionals who participated in the pilot test were not included in the formal Delphi sample.

The modified Delphi process comprised two rounds. The first Delphi round was conducted from August to October 2022, and the second round from January to March 2023. In the first round, experts rated the relevance of the eight candidate clinical parameters using a four-point Likert scale (irrelevant, somewhat relevant, relevant, and very relevant). A four-point scale was adopted to avoid neutral responses and encourage definitive expert judgments. The first-round questionnaire also included an open-ended field in which experts could suggest additional clinical parameters not included among the eight candidate items. Suggestions were reviewed for potential inclusion as additional candidate items in the second Delphi round. For each candidate item, the Item-level Content Validity Index (I-CVI) was calculated by dividing the number of experts rating the item as relevant or very relevant by the total number of experts included in the first-round analysis (n = 53). No Scale-level CVI (S-CVI) was calculated. The approved study protocol specified agreement greater than 60% (I-CVI > 0.60) on the Likert scale as the criterion for item progression to the second Delphi round. Because methodological recommendations suggest an I-CVI of ≥ 0.78 as evidence of good content validity when multiple experts are involved,^10^ a post hoc sensitivity assessment using this threshold was also performed.

To ensure consistency of evaluation, experts received instructions regarding the timing of assessment for each clinical parameter. The first five items were assessed 2 minutes after administration of the hypnotic and neuromuscular blocking agent and before electrical stimulus delivery; the sixth and seventh were assessed immediately after seizure termination, and the eighth following recovery from anesthesia.

All experts who completed the first Delphi round were invited to participate in the second round; no new experts were recruited. Aggregated first-round results were not formally fed back to participants before the second round. The eight candidate items were rated on a numerical scale from 0 to 10, with higher scores indicating greater perceived relevance. The questionnaires administered in both rounds of the modified Delphi process are provided in Supplementary Appendix S1. Exploratory Principal Component Analysis (PCA) was performed on expert item-relevance ratings to explore the covariance structure of expert judgments and to support preliminary item refinement. Because the analysis was based on expert ratings rather than ECTAS scores obtained from patients, it was not interpreted as evidence of the dimensional structure or construct validity of the clinical instrument. Sampling adequacy and matrix factorability were assessed using the Kaiser-Meyer-Olkin (KMO) measure and Bartlett’s test of sphericity. Given the ordinal nature of the expert ratings and the modest sample size, the PCA was considered exploratory. The internal consistency of expert item-relevance ratings was explored using Cronbach's alpha. This analysis characterized the consistency of experts' ratings of the candidate items and was not interpreted as evidence of the internal consistency or reliability of ECTAS when applied clinically. Statistical analyses were performed using IBM SPSS Statistics version 29.0. The preliminary four-item version of the instrument was established by integrating expert-based content validity findings, exploratory analysis of expert item-relevance ratings, and clinical judgment by the research team regarding the intended pre-stimulation assessment.

A total of 53 experts participated in the first Delphi round, including 42 anesthesiologists (79.2%) and 11 psychiatrists (20.8%). Thirty-two participants (60.4%) reported more than 10 years of experience with ECT. Additional characteristics of the first-round expert panel are presented in Supplementary Table S1.

In the first Delphi round, apparent immobility after succinylcholine-induced fasciculations (I-1) achieved the highest Content Validity Index (I-CVI = 0.925), whereas seizure duration of at least 20 seconds (I-7) had the lowest (I-CVI = 0.736). All eight candidate items exceeded the protocol-defined criterion of > 60% agreement and therefore progressed to the second Delphi round. In the post hoc sensitivity assessment using an I-CVI threshold of ≥ 0.78, seven of the eight items met this more stringent criterion. I-7 (seizure duration ≥ 20 seconds) had an I-CVI of 0.736. The complete content validity results are presented in Table 1.

**Table 1:** Expert relevance ratings and Item-level Content Validity Index (I-CVI) for the eight candidate clinical parameters evaluated in the first Delphi round.

Candidate clinical parameter	Relevant or very relevant, n (%)	I-CVI
Apparent immobility after the fasciculations resulting from the administration of succinylcholine ceased	49 (92.5)	0.925
Lack of eye opening to verbal commands	43 (81.1)	0.811
Absence of spontaneous breathing (apnea)	46 (86.8)	0.868
Abolition of the corneal-palpebral reflex	45 (84.9)	0.849
Reduced muscle strength (Medical Research Council grade < 2)	42 (79.2)	0.792
Clonic jerks absent (except in the limb with a tourniquet, when used) or present at a low intensity	48 (90.6)	0.906
At least 20 seconds of induced seizures	39 (73.6)	0.736
Memory of the event and/or perception of breathlessness on awakening	48 (90.6)	0.906

I-CVI, Item-level Content Validity Index. I-CVI was calculated as the proportion of experts rating each item as relevant or very relevant.

The open-ended field generated additional suggestions from both professional groups. Anesthesiologists suggested consciousness monitoring, ease of mouthguard placement, visual assessment of the baseline electroencephalogram, total time in the procedure room and post-anesthesia recovery, reductions in heart rate and blood pressure after loss of consciousness, and absence of masseter contraction. Psychiatrists suggested reduction in heart rate and post-anesthesia recovery time. After review by the research team, none of these suggestions were incorporated as additional candidate items for the second Delphi round because they either involved instrumental monitoring, reflected procedural or recovery outcomes rather than pre-stimulation bedside findings, or were considered potentially dependent on the anesthetic technique.

Of the 53 experts who participated in the first round, 38 completed the second round, corresponding to a retention rate of 71.7%. The second-round panel comprised 29 anesthesiologists (76.3%) and nine psychiatrists (23.7%). Item-level relevance ratings for the eight candidate items are presented as median and interquartile range in Supplementary Table S2. Expert item-relevance ratings across the eight candidate items yielded a Cronbach's α of 0.84. Exploratory Principal Component Analysis (PCA) of these ratings showed that the first principal component accounted for 48.0% of the total variance. For the eight-item analysis, KMO was 0.722 and Bartlett’s test of sphericity was significant (χ^2^(28) = 132.995, p < 0.001).

Following the second Delphi round, investigator-led refinement was undertaken to align the candidate items with the intended pre-stimulation assessment. I-6 and I-7 were excluded because they can only be assessed after electrical stimulus delivery, whereas I-3 was excluded because it was considered to provide overlapping clinical information with apparent immobility (I-1). I-8 was retained as a separate complementary post-anesthetic parameter rather than as part of the pre-stimulation scale. This investigator-led refinement, informed by the expert relevance ratings, exploratory PCA, and clinical judgment, resulted in the preliminary four-item configuration comprising I-1, I-2, I-4, and I-5. The resulting four-item configuration was not submitted to a third Delphi round or other subsequent expert consensus procedure.

A second exploratory PCA was performed on expert ratings of the four retained pre-stimulation items (I-1, I-2, I-4, and I-5). The first principal component accounted for 63.8% of the total variance in expert ratings. For the four-item analysis, KMO was 0.700 and Bartlett’s test was also significant (χ^2^(6) = 54.157, p < 0.001). Detailed eigenvalues, variance explained, cumulative variance, and component loadings for both PCA analyses are presented in Supplementary Table S3.

The preliminary instrument was designated the Electroconvulsive Therapy Anesthesia Scale (ECTAS) and comprises four pre-stimulation clinical parameters intended to structure the bedside assessment of anesthetic readiness before electrical stimulus delivery (Table 2). Item I-8, addressing memory of the procedure and/or perceived dyspnea upon awakening, received one of the highest content validity ratings but represents a post-anesthetic outcome rather than a pre-stimulation finding. It was therefore retained for separate consideration as a complementary post-anesthetic parameter and is not part of the four-item pre-stimulation ECTAS. Its potential clinical utility, including whether it may inform anesthetic management in subsequent ECT sessions, requires prospective evaluation. No readiness cutoff was defined at this stage because the study did not include patient-level clinical validation or outcome-based assessment from which a clinically meaningful threshold could be derived.

**Table 2:** Preliminary clinical parameters retained for the Electroconvulsive Therapy Anesthesia Scale (ECTAS).

Retained pre-stimulation clinical parameter
Apparent immobility after the fasciculations resulting from the administration of succinylcholine ceased
Absence of eye opening to verbal commands
Abolition of the corneal-palpebral reflex
Reduced muscle strength (Medical Research Council grade < 2)

*No item weighting or readiness cutoff has been established.

The present study describes the preliminary development of a structured bedside instrument for assessing anesthetic readiness before electrical stimulus delivery during Electroconvulsive Therapy (ECT) and provides preliminary expert-based evidence of content validity. ECTAS was designed to structure bedside clinical assessment based on physical examination and routine clinical observations and is not intended to replace quantitative neuromuscular monitoring.

The four retained pre-stimulation items encompass clinically distinct domains of anesthetic readiness. Absence of eye opening to verbal commands and abolition of the corneal-palpebral reflex were conceptualized as primarily related to hypnotic effect, whereas apparent immobility and reduced muscle strength were conceptualized as primarily related to attenuation of motor response and neuromuscular blockade. These domains are physiologically distinct and should not be interpreted as interchangeable. In particular, immobility resulting from neuromuscular blockade cannot be considered evidence of adequate hypnosis. Their inclusion within a structured assessment reflects expert judgment regarding their clinical relevance before electrical stimulation rather than evidence that they measure a single latent construct.

Although these clinical parameters may be assessed individually by anesthesiologists, their interpretation may be subjective and non-standardized. A structured assessment may facilitate standardized clinical documentation, training, and a common framework for future clinical studies. However, whether ECTAS improves consistency between observers or clinical decision-making cannot be established from the present study and requires prospective evaluation.

Exploratory PCA was used to examine the covariance structure of expert item-relevance ratings and to assist preliminary instrument refinement. The first principal component accounted for 48.0% of the variance across the eight candidate items and 63.8% across the four retained pre-stimulation items. However, because these analyses were based exclusively on expert judgments rather than patient-level ECTAS measurements, they should not be interpreted as evidence of unidimensionality, construct validity, or the dimensional structure of the clinical instrument. Similarly, the reported Cronbach's alpha was calculated from expert item-relevance ratings and therefore describes the consistency of expert judgments rather than the internal consistency or reliability of ECTAS when applied clinically. The dimensional structure and measurement properties of ECTAS require prospective evaluation using patient-level data.

The present expert-based development study also does not provide clinical evidence to support equal weighting of the four retained items or a specific readiness threshold. Because the items represent distinct physiological domains, different combinations of positive findings may correspond to clinically different anesthetic states. Therefore, no cutoff for anesthetic readiness is proposed at this stage. The clinical interpretation of the individual items, the appropriateness of any weighting scheme, and whether a clinically meaningful threshold can be defined should be investigated prospectively using patient-level data.

Although I-8 is not part of the four-item pre-stimulation ECTAS, it was retained separately as a complementary post-anesthetic parameter. Its potential utility for informing anesthetic management in subsequent ECT sessions requires prospective evaluation.

This study has several limitations. The Delphi process, Cronbach's alpha analysis, and PCA were based on expert ratings rather than patient-level clinical observations and therefore provide no evidence regarding the clinical reliability, construct or criterion validity, responsiveness, diagnostic performance, or safety of ECTAS. Inter-rater reliability was not evaluated and represents an essential next step before clinical implementation. Attrition between Delphi rounds represents an additional limitation: 38 of the 53 first-round participants (71.7%) completed the second round, and the possibility that nonrespondents differed systematically from continuing participants cannot be excluded. Furthermore, the Delphi panel consisted exclusively of Brazilian specialists, which may limit the generalizability of the expert-based findings.

An additional limitation concerns the operational definition of I-5. The item was evaluated by the expert panel as “reduction in muscle strength (MRC grade < 2)”. However, the MRC grading system depends on voluntary motor effort, limiting its applicability after induction of general anesthesia and neuromuscular blockade. The original questionnaire did not specify the muscle group assessed, the standardized motor command used, or the approach to scoring muscle strength in patients unable to follow commands. Similarly, the technique used to elicit and assess the corneal-palpebral reflex was not prospectively standardized. Because these operational details were not prospectively defined, no retrospective definitions were assigned. These items therefore require further operational refinement and subsequent content assessment before clinical validation.

Prospective patient-level studies should evaluate the inter-rater reliability of ECTAS, construct and criterion validity, responsiveness, and, where applicable, test-retest reliability. Future validation should also examine relationships with prespecified domain-specific external measures and clinically relevant procedural and recovery outcomes, with the selection of appropriate comparators defined according to the measurement property and physiological domain under investigation.

In conclusion, ECTAS is a preliminary structured bedside assessment comprising four pre-stimulation clinical parameters retained through a modified Delphi process and expert-based content validity assessment. The present findings support the relevance of these retained parameters but do not establish the clinical validity, reliability, dimensional structure, diagnostic performance, or readiness threshold of ECTAS. Prospective patient-level validation is required before its use for clinical decision-making can be recommended.

## Data availability statement

The anonymized data underlying the findings of this study, together with the data dictionary and statistical analysis materials, are publicly available in Zenodo at https://doi.org/10.5281/zenodo.22648934.

## Declaration of generative AI and AI-assisted technologies in the writing process

During the preparation and revision of this manuscript, the authors used ChatGPT (OpenAI) and Claude (Anthropic) to assist with language editing, improvement of clarity and readability, and organization of the revised text. These tools were not used for data collection or primary statistical analysis. All methodological decisions, interpretation of the results, and final manuscript content were reviewed and approved by the authors, who take full responsibility for the work.

## Authors’ contributions

MVGM, JOBN, and JBSG participated in the conception and design of the study, writing and critical review of the manuscript, and approval of the final version to be submitted; MVGM, GMNG, BBP, MALE and JCOS participated in the collection, analysis, and interpretation of the data.

## Funding

This research did not receive any specific grant from funding agencies.

## Ethical approval number

5.456.362.

## Conflicts of interest

The authors declare no conflicts of interest.

## Appendix Supplementary materials

Image, application 1

## Footnotes

Supplementary material associated with this article can be found in the online version at doi:10.1016/j.bjane.2026.844842.
