# Supplementary material for: Electroconvulsive Therapy Anesthesia Scale (ECTAS): development and preliminary expert-based evidence of content validity
Source: Braz J Anesthesiol. 2026 Oct 9;77(1):844842. doi: 10.1016/j.bjane.2026.844842 (PMC13738276; doi:10.1016/j.bjane.2026.844842)
Supplement: Supplementary file 1 [file mmc1.docx]

**BJAN-D-26-00613_Supplementary Material**

**Supplementary Appendix S1. Questionnaires administered during the modified Delphi process**

Note: The questionnaires below reproduce the wording administered to participants during the original study. Terminology used in the current manuscript was subsequently refined to distinguish the broader concept of anesthetic quality from the intended pre-stimulation assessment of anesthetic readiness.

**Part A. First-Round Delphi Questionnaire**

Questionnaire Introduction

Objective: To evaluate the relevance of clinical parameters for assessing the quality of anesthesia during electroconvulsive therapy (ECT) procedures, based on practitioners’ clinical experience.

General Instructions:

• Please respond based on your clinical experience with ECT (minimum of 2 years).

• In Section 1 (Items 1–8), following the demographic information, please assess the relevance of each clinical parameter using the following scale: Irrelevant (1), Somewhat Relevant (2), Relevant (3), and Very Relevant (4).

• The first five parameters should be assessed 2 minutes after administration of the hypnotic and neuromuscular blocking agent.

• Absence of tonic-clonic movements and seizure duration should be assessed immediately after seizure termination.

• Recall of the event and/or perception of breathlessness should be assessed based on patient self-report after full recovery from anesthesia.

• Section 2 consists of an open-ended field for additional comments or suggestions.

• Estimated completion time: 5 minutes.

Demographic Information:

• Specialty: Anesthesiologist / Psychiatrist

• Years of experience with ECT: < 5 / 5–10 / 11–15 / 16–20 / > 20

• Highest academic degree: Specialist Degree / Master’s Degree / PhD (Doctorate)

• Primary professional focus: Clinical Practice / Teaching / Research

Section 1: Clinical Parameter Relevance Assessment

| Item | Clinical parameter | Irrelevant | Somewhat Relevant | Relevant | Very relevant |
| --- | --- | --- | --- | --- | --- |
| 1 | Apparent immobility after the fasciculations resulting from the administration of succinylcholine ceased. |  |  |  |  |
| 2 | Lack of eye opening to verbal commands. |  |  |  |  |
| 3 | Absence of spontaneous breathing (apnea). |  |  |  |  |
| 4 | Abolition of the corneal-palpebral reflex. |  |  |  |  |
| 5 | Reduced muscle strength (Medical Research Council grade < 2). |  |  |  |  |
| 6 | Clonic jerks absent (except in the limb with a tourniquet, when used) or present at a low intensity. |  |  |  |  |
| 7 | At least 20 seconds of induced seizures. |  |  |  |  |
| 8 | Memory of the event and/or perception of breathlessness on awakening. |  |  |  |  |

Section 2: Additional Suggestions

Please suggest any additional clinical parameters relevant to assessing the quality of anesthesia during ECT that were not listed above. (Open-text field)

Closing Remarks

• Thank you for your participation.

**Part B. Second-Round Delphi Questionnaire**

Questionnaire Introduction

Objective: To reassess the relevance of the clinical parameters for evaluating the quality of anesthesia during electroconvulsive therapy (ECT).

General Instructions:

• This second-round questionnaire was administered only to experts who had participated in the first Delphi round.

• Please rate the relevance of each clinical parameter on a numerical scale from 0 to 10, with higher scores indicating greater perceived relevance.

• The first five parameters should be assessed 2 minutes after administration of the hypnotic and neuromuscular blocking agent.

• Absence of tonic-clonic movements and seizure duration should be assessed immediately after seizure termination.

• Recall of the event or perception of breathlessness should be assessed based on patient self-report after full recovery from anesthesia.

Clinical Parameter Relevance Assessment

1. Apparent immobility after the fasciculations resulting from the administration of succinylcholine ceased.

Rating: 0–10

2. Lack of eye opening to verbal commands.

Rating: 0–10

3. Absence of spontaneous breathing (apnea).

Rating: 0–10

4. Abolition of the corneal-palpebral reflex.

Rating: 0–10

5. Reduced muscle strength (Medical Research Council grade < 2).

Rating: 0–10

6. Clonic jerks absent (except in the limb with a tourniquet, when used) or present at a low intensity.

Rating: 0–10

7. At least 20 seconds of induced seizures.

Rating: 0–10

8. Memory of the event and/or perception of breathlessness on awakening.

Rating: 0–10

Closing Remarks:

Thank you for your participation.

**Supplementary Table S1** Characteristics of the expert panel participating in the first Delphi round (n = 53).

| **Variable** | **Category** | **n** | **%** |
| --- | --- | --- | --- |
| Specialty | Anesthesiologist | 42 | 79.2 |
|  |  |  |  |
|  | Psychiatrist | 11 | 20.8 |
|  |  |  |  |
| Years of experience with ECT | < 5 years | 9 | 17.0 |
|  | 5–10 years | 12 | 22.6 |
|  |  |  |  |
|  | 11–15 years | 23 | 43.4 |
|  |  |  |  |
|  | 16–20 years | 7 | 13.2 |
|  |  |  |  |
|  | > 20 years | 2 | 3.8 |
|  |  |  |  |
| Highest academic degree | Specialist degree | 29 | 54.7 |
|  |  |  |  |
|  | Master’s degree | 9 | 17.0 |
|  | Doctorate | 15 | 28.3 |
| Primary professional focus | Clinical practice | 32 | 60.4 |
|  | Teaching | 17 | 32.1 |
|  | Research | 4 | 7.5 |

**Supplementary Table S2** Second-round expert relevance ratings for the eight candidate items (n = 38).

| **Item** | **Candidate clinical parameter** | **Median (Q1–Q3)** |
| --- | --- | --- |
| I-1 | Apparent immobility after succinylcholine-induced fasciculations had ceased | 8.5 (7–10) |
| I-2 | Absence of eye opening in response to verbal commands | 9 (7.75–10) |
| I-3 | Apnea | 8 (6–10) |
| I-4 | Abolition of the corneal-palpebral reflex | 8 (6–10) |
| I-5 | Reduced muscle strength (MRC grade < 2) | 9 (8–10) |
| I-6 | Absent or low-intensity tonic-clonic movements | 8 (8–10) |
| I-7 | Seizure duration ≥ 20 seconds | 8 (6–10) |
| I-8 | Memory of the event and/or breathlessness upon awakening | 10 (9–10) |

Note: Values are presented as median (25th–75th percentile). Ratings ranged from 0 to 10, with higher scores indicating greater perceived relevance.

**Supplementary Table S3** Exploratory principal component analyses of expert item-relevance ratings.

**A. Eight-item analysis (n = 38)**

| **Measure** | **Result** |
| --- | --- |
| Kaiser–Meyer–Olkin (KMO) measure | 0.722 |
| Bartlett’s test of sphericity | χ²(28) = 132.995, *p* < 0.001 |

| **Component** | **Eigenvalue** | **Variance explained (%)** | **Cumulative variance (%)** |
| --- | --- | --- | --- |
| PC1 | 3.842 | 48.02 | 48.02 |
| PC2 | 1.403 | 17.53 | 65.55 |
| PC3 | 0.852 | 10.65 | 76.21 |
| PC4 | 0.752 | 9.40 | 85.60 |
| PC5 | 0.413 | 5.17 | 90.77 |
| PC6 | 0.306 | 3.83 | 94.60 |
| PC7 | 0.275 | 3.44 | 98.04 |
| PC8 | 0.157 | 1.96 | 100.00 |

**Component loadings**

| **Item** | **PC1** | **PC2** | **PC3** | **PC4** | **PC5** | **PC6** | **PC7** | **PC8** |
| --- | --- | --- | --- | --- | --- | --- | --- | --- |
| I-1 | -0.864 | -0.106 | 0.048 | 0.021 | -0.360 | 0.263 | 0.046 | 0.197 |
| I-2 | -0.625 | -0.454 | 0.374 | 0.322 | 0.320 | 0.184 | -0.145 | -0.048 |
| I-3 | -0.689 | -0.390 | -0.494 | -0.064 | 0.176 | -0.229 | -0.113 | 0.170 |
| I-4 | -0.719 | -0.412 | -0.078 | -0.452 | 0.034 | 0.050 | 0.256 | -0.184 |
| I-5 | -0.819 | 0.170 | -0.217 | 0.304 | -0.265 | -0.109 | -0.172 | -0.220 |
| I-6 | -0.508 | 0.635 | 0.038 | -0.489 | 0.138 | 0.127 | -0.250 | -0.001 |
| I-7 | -0.693 | 0.190 | 0.595 | -0.043 | -0.030 | -0.339 | 0.090 | 0.061 |
| I-8 | -0.551 | 0.628 | -0.239 | 0.325 | 0.243 | 0.072 | 0.272 | 0.025 |

**B. Four-item analysis (I-1, I-2, I-4, and I-5; n = 38)**

| **Measure** | | | **Result** | | |
| --- | --- | --- | --- | --- | --- |
| Kaiser–Meyer–Olkin (KMO) measure | | | 0.700 | | |
| Bartlett’s test of sphericity | | | χ²(6) = 54.157, *p* < 0.001 | | |
| **Component** | **Eigenvalue** | **Variance explained (%)** | | **Cumulative variance (%)** |  |
| PC1 | 2.554 | 63.84 | | 63.84 |  |
| PC2 | 0.669 | 16.73 | | 80.57 |  |
| PC3 | 0.554 | 13.85 | | 94.42 |  |
| PC4 | 0.223 | 5.58 | | 100.00 |  |

**Component loadings**

| **Item** | **PC1** | **PC2** | **PC3** | **PC4** |
| --- | --- | --- | --- | --- |
| I-1 | -0.910 | -0.162 | -0.078 | -0.374 |
| I-2 | -0.726 | 0.456 | 0.512 | 0.046 |
| I-4 | -0.770 | 0.335 | -0.519 | 0.159 |
| I-5 | -0.778 | -0.568 | 0.127 | 0.237 |

Note: PCA, principal component analysis; PC, principal component; KMO, Kaiser–Meyer–Olkin measure. PCA was performed on the correlation matrix of expert item-relevance ratings. Component signs are mathematically arbitrary and do not affect the PCA solution.
